# The Crosstalk between Intestinal Epithelial Cells and Mast Cells Is Modulated by the Probiotic Supplementation in Co-Culture Models

**DOI:** 10.3390/ijms24044157

**Published:** 2023-02-19

**Authors:** Raffaella di Vito, Alessia Di Mezza, Carmela Conte, Giovanna Traina

**Affiliations:** Department of Pharmaceutical Sciences, University of Perugia, Via Romana, 06126 Perugia, Italy

**Keywords:** mast cells, HMC-1.2, Caco-2, HT-29, Transwell co-culture model, Serobioma, probiotics, immunoregulation, intestinal barrier

## Abstract

The intestinal epithelium constitutes a selectively permeable barrier between the internal and external environment that allows the absorption of nutrients, electrolytes, and water, as well as an effective defense against intraluminal bacteria, toxins, and potentially antigenic material. Experimental evidence suggest that intestinal inflammation is critically dependent on an imbalance of homeostasis between the gut microbiota and the mucosal immune system. In this context, mast cells play a crucial role. The intake of specific probiotic strains can prevent the development of gut inflammatory markers and activation of the immune system. Here, the effect of a probiotic formulation containing *L. rhamnosus* LR 32, *B. lactis* BL04, and *B. longum* BB 536 on intestinal epithelial cells and mast cells was investigated. To mimic the natural host compartmentalization, Transwell co-culture models were set up. Co-cultures of intestinal epithelial cells interfaced with the human mast cell line HMC-1.2 in the basolateral chamber were challenged with lipopolysaccharide (LPS), and then treated with probiotics. In the HT29/HMC-1.2 co-culture, the probiotic formulation was able to counteract the LPS-induced release of interleukin 6 from HMC-1.2, and was effective in preserving the epithelial barrier integrity in the HT29/Caco-2/ HMC-1.2 co-culture. The results suggest the potential therapeutic effect of the probiotic formulation.

## 1. Introduction

The human gastrointestinal (GI) tract represents the largest interfaces between the host and the external environment [1]. Moreover, it contains a higher number of immune cells than other body compartment [2].

The first line of defense against pathogens is constituted of the intestinal epithelium. It is a single layer of epithelial cells arranged into villi and crypts. Together with the mucus layer, it forms the primary physical and biochemical barrier that separates the intestinal lumen’s content from the cells located beneath the lamina propria [3]. Most intestinal lumen-contacting cells are absorbent enterocytes with digestive and metabolic functions. In addition, there are secretory epithelial cells, including enteroendocrine cells, Paneth cells, and goblet cells. While enteroendocrine cells secrete hormones that regulate digestive functions, Paneth cells produce antimicrobial peptides that help in preventing microbial infection, and goblet cells secrete highly glycosylated mucins into the intestinal lumen, with a protective role against microbial encroachment [3]. Moreover, intraepithelial lymphocytes, the lamina propria, and the gut-associated lymphoid tissue all contribute to the host’s defence [2]. Of note, the gut microbiota also represent an important physical barrier against ingested pathogenic bacteria through competitive exclusion, and resistance to colonization [4].

Enterocytes can act as antigen-presenting cells, as they express the class II major histocompatibility complex on their basolateral membrane. In addition, they can regulate T cell response in the intestinal mucosa [5]. Most of the immune cells are present at lamina propria level and include CD4^+^ T-cells, CD8^+^ T-cells, IgA-producing plasma cells, dendritic cells, macrophages, mast cells (MCs), and eosinophils, as well as innate lymphoid cells [2].

MCs are innate immunity cells, widely distributed in tissues, strategically located at the interface with the external environment, where they act as sentinels toward invading antigens. On the other hand, they play pathogenic roles in many allergic and inflammatory reactions [6]. In the gut, MCs are important orchestrators of homeostasis, and their functions span a wide range of strategic aspects, from pathogen clearance to the maintenance of the intestinal epithelium’s integrity [7]. MCs originate from hematopoietic stem cells, leave the bone marrow in an immature form, and complete their differentiation in tissues [8,9]. The development of mature granules is activated by the binding between the tyrosine-protein kinase receptor on the MC progenitor’s surface and the stem cell factor, expressed from fibroblasts, stromal cells, and endothelial cells [10]. MCs are able to produce, store, and release a vast collection of chemical mediators including cytokines [11]. The composition of MC granules shows heterogeneity depending on their tissue localization [12]. Mediators contained inside the granules can be preformed and de novo synthesized. Histamine, heparin, TNF-α, and serotonin are preformed mediators, released a few seconds after stimulation. Conversely, lipid-derived mediators and some cytokines, including interleukin (IL)-1β, IL-4, IL-6, and IL-10 are produced after cell stimulation and are released within minutes or hours [10,13].

MCs are located in both the small and the large intestines, and appear to be functionally different along the GI tract. They respond to microbial antigens via toll-like receptors (TLRs) on the membrane surface, initiating an innate immune response that culminates with the release of the cytokines. The more abundant colonization by bacteria in the colon than in the small intestine could explain the higher expression of the TLRs in the colon-resident MCs [11]. Several studies have demonstrated that TLR2 and TLR4 are involved in bacteria-induced MC activation by peptidoglycan and lipopolysaccharide (LPS), respectively. While TLR2 mediated response consists in degranulation followed by cytokine release, TLR4 activates the cytokine release without degranulation [13,14].

Emerging evidence supports a key role of MCs in mediating the crosstalk between host and microbiota through the mutual modulation of the activation status, which in turn affects the intestinal epithelial barrier stability [15,16].

Gut dysbiosis impairs the immune system homeostasis and induces a non-immunogenic hyperinflammatory response that precedes several pathological conditions [17]. Selected probiotic strains have been shown to possess beneficial properties, including antigenotoxic, antioxidant, and anti-inflammatory activities [18,19,20,21]. Therefore, supplementation with specific probiotic strains is considered a preventive/therapeutic strategy for dysbiosis management and immune homeostasis [22], that could also contribute to MC stabilization [6,23].

In a previous study, the immunomodulatory effects of a commercially available probiotic formulation, namely Serobioma^®^ (Bromatech S.r.l., Milan, Italy), was demonstrated in an in vitro Transwell model of HT-29 intestinal cells interfaced with both THP-1-derived inflammatory macrophages type 1, and with ex vivo human monocyte-derived macrophages [24]. In the present study, the same probiotic formulation was employed in order to investigate the immunomodulatory potential in two different co-culture models. In particular, we used HT-29 cells and a co-culture of Caco-2/HT-29 cells, the latter considered the best model to mimic the human intestinal epithelium in vitro [25,26]. In both cases, the epithelial layer was interfaced with a human MC line, namely HMC-1.2, in the basolateral compartment. Despite being immature, HMC-1.2 cells exhibit a phenotype similar to the human tissue MCs and a high proliferation rate. These features make HMC-1.2 particularly attractive and widely used [27].

The cells were challenged with the LPS proinflammatory stimulus and then treated with the Serobioma multi-strain probiotic formulation. The transepithelial electrical resistance (TEER) measurement was used to evaluate the barrier function of the cellular layers [28], while the inflammatory response was assessed by measuring cytokine production.

## 2. Results

### 2.1. TEER Measurement

**Figure 1:**
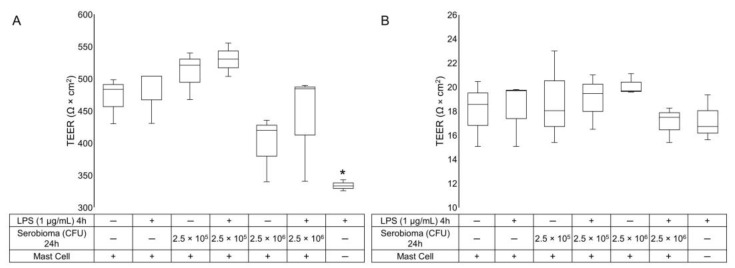
TEER measurement. (**A**) Caco-2/HT-29 co-culture and (**B**) HT-29 confluent cells were challenged with LPS for 4 h and then treated with the probiotic mixture for a further 24 h in the presence of HMC-1.2, in the basolateral compartment. Statistical analysis: one-way ANOVA followed by Dunnett’s post hoc, * *p*-value < 0.05 (*n* = 3).

**Figure 2:**
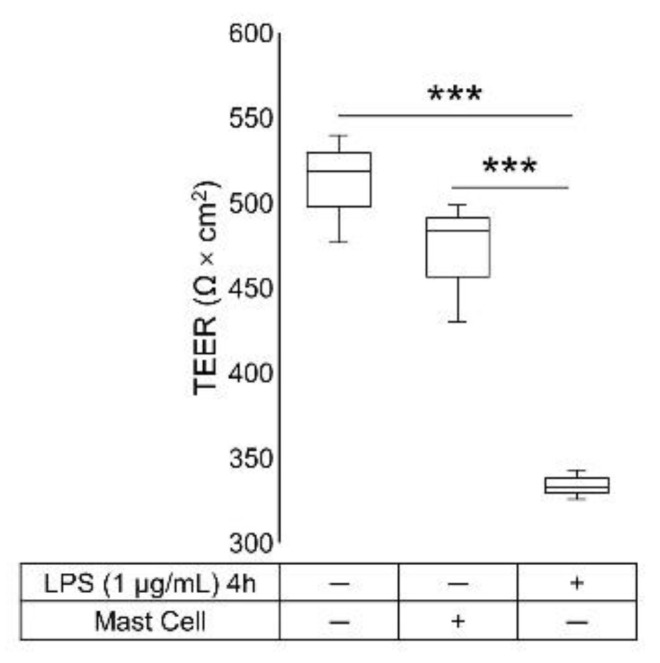
TEER measurement of Caco-2/HT-29 co-culture in the presence or not of HMC-1.2 in the basal compartment and after 4 h LPS stimulation. Statistical analysis: one-way ANOVA followed by Bonferroni’s post hoc, *** *p*-value < 0.001 (*n* = 3).

In order to evaluate the integrity of the epithelial barrier, a TEER measurement was performed. The mean TEER value in the HT-29/Caco-2 co-culture was 470.54 ± 20.79 Ω × cm^2^, and treatment with LPS for 4 h, in the absence of HMC-1.2, induced a significant reduction in the TEER values (*p*-value = 0.013). The treatment with the probiotic formulation did not affect the integrity of the epithelial barrier (Figure 1A). Notably, the presence of HMC-1.2, in the absence of probiotics, did not alter the basal TEER values of the HT-29/Caco-2 co-culture (Figure 2).

On the contrary, the mean of the TEER values for the HT-29 model was 18.03 ± 1.59 Ω × cm^2^, and no significant differences were observed in the presence of the probiotic formulation or LPS (Figure 1B).

### 2.2. ELISA Assay

**Figure 3:**
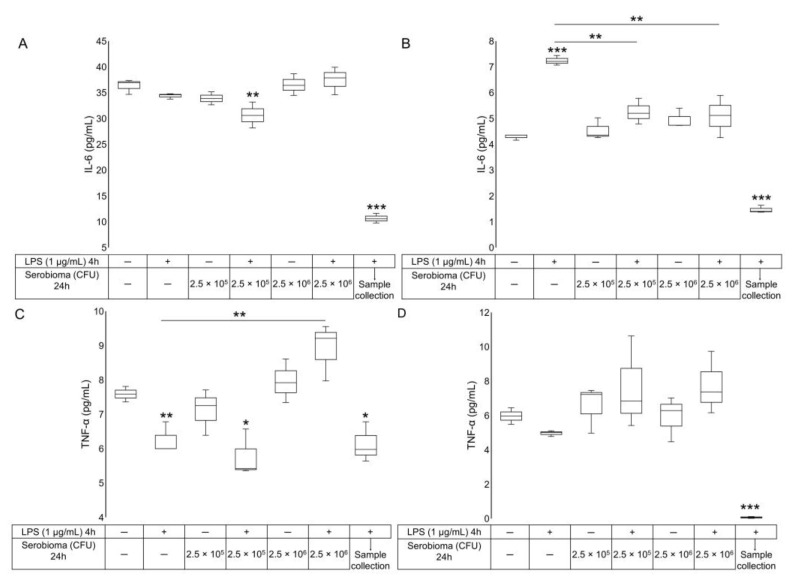
HMC-1.2 cytokine release after LPS/Serobioma treatment. HMC-1.2 were interfaced with (**A**,**C**) the Caco-2/HT-29 co-culture intestinal cells or (**B**,**D**) confluent HT-29 cells. Statistical analysis: one-way ANOVA followed by Dunnett’s post hoc with respect to the untreated control. Cells pre-incubated with LPS and then with Serobioma were also compared with the LPS-treated group. * *p*-value < 0.05, ** *p*-value < 0.01, *** *p*-value < 0.001 (*n* = 3).

In order to determine the effect of the probiotic formulation on the proinflammatory cytokines release, an ELISA assay was employed. In the model, comprised of HMC-1.2 interfaced with the Caco-2/HT-29 co-culture, the LPS did not induce any significant changes in IL-6 release (Figure 3A). On the contrary, the treatment with LPS for 4 h and then with Serobioma, at a 1:1 cell/colony forming units (CFU) ratio, for a further 24 h, caused a decrease in IL-6 levels (*p*-value = 0.009). Again, compared with the untreated control, HMC-1.2 exposed to LPS for 4 h, without replacement with complete medium (recovery time), released a lower amount of IL-6 compared with untreated cells (*p*-value < 0.0001) (Figure 3A).

The treatment of HT-29/HMC-1.2 with LPS for 4 h, followed by 24 h with complete medium, produced a significant increase in IL-6 release (*p*-value < 0.0001, Figure 3B). Conversely, HMC-1.2 exposed to LPS for 4 h with no recovery time, released a significantly lower amount of IL-6 with respect to untreated cells. Notably, the probiotic formulation is able to prevent the LPS-induced release of IL-6 from the MCs at all the concentrations tested (*p*-value = 0.009 and 0.006 for samples stimulated for 4 h with LPS and then exposed to probiotic at 1:1 and 1:10 cell/CFU ratios, respectively) (Figure 3B).

Interestingly, an 8-fold higher amount of released IL-6 was observed when HMC-1.2 was interfaced with the HT-29/Caco-2 co-culture (36.30 ± 0.86 pg/mL) (Figure 3A), compared to MCs interfaced with HT-29 alone (4.29 ± 0.06 pg/mL) (Figure 3B). To exclude the possibility that the Caco-2 cells generated IL-6, the same experimental procedure was performed in Caco-2/HT-29 co-cultures in the absence of HMC-1.2. The results demonstrated that there was no release of IL-6.

Finally, another marker of inflammation, the TNF-α, was measured. Unexpectedly, in the Caco-2/HT-29/HMC-1.2 model, the LPS treatment induced a significant decrease in TNF-α release from HMC-1.2 cells (*p*-value = 0.010) (Figure 3C). These levels of TNF-α are comparable with the sample pre-treated with LPS and then exposed to probiotic formulation at a 1:1 cell/CFU ratio, and with the 4 h LPS-treated sample without the recovery time. Importantly, the post-treatment with bacteria at a 1:10 cell/CFU ratio seems to counteract the decrease in TNF-α caused by LPS (*p*-value = 0.005 compared to the LPS-treated sample, Figure 3C). On the contrary, in the HT-29/HMC-1.2 co-culture, no TNF-α release was observed after 4 h of LPS exposure (Figure 3D), and no other significance was highlighted.

IL-1β and IL-10 were not detected with the ELISA assay.

### 2.3. RT-qPCR

**Figure 4:**
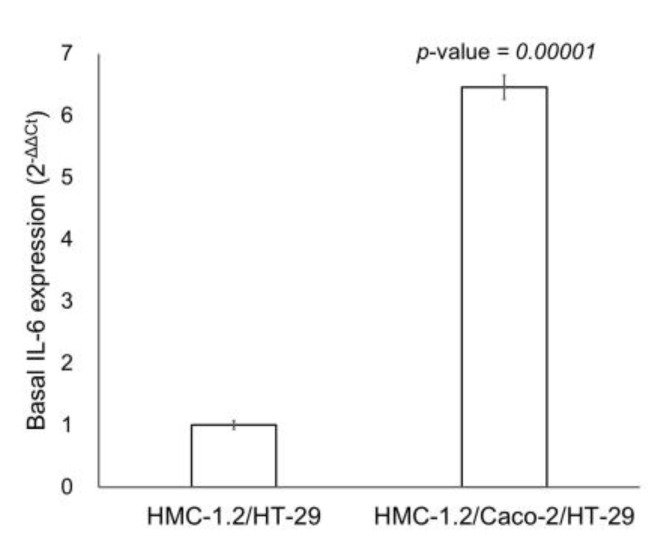
*IL-6* gene expression analysed by RT-qPCR in HMC-1.2 interfaced with HT-29 and with the Caco-2/HT-29 co-culture. Results are reported as mean ± SEM of three independent experiments. Statistical analysis: Student’s *t* test.

The levels of IL-6 mRNA in HMC-1.2 was analyzed by RT-qPCR. As shown in Figure 4, when cells were interfaced with the Caco-2/HT-29 co-culture, about a 7-fold increase in the expression of *IL-6* was observed (*p*-value = 0.00001).

### 2.4. DAPI-Staining

**Figure 5:**
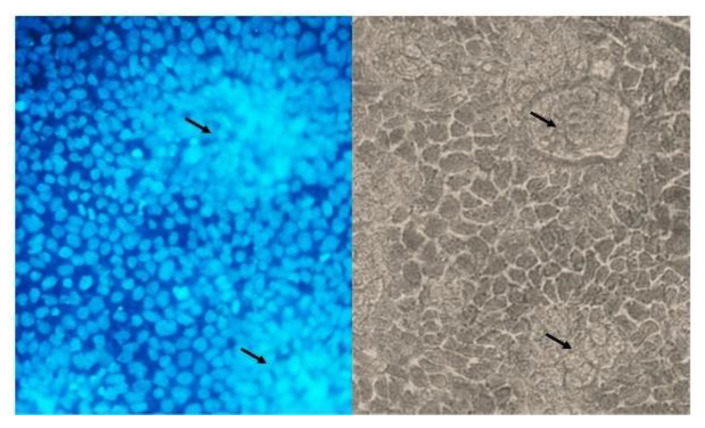
Microphotograph of the Caco-2/HT-29 co-culture seeded on the Transwell insert at a 9:1 ratio and cultured for 18 days. Morphological features were examined with DAPI staining (on the **left**). Black arrows indicate HT-29 clusters inside the differentiated Caco-2 cell monolayer, 20× magnification.

Microscopic analysis with DAPI staining was used to examine the morphology of the Caco-2/HT-29 co-culture. Figure 5 shows that HT-29 cells form clusters inside the Caco-2 monolayer.

## 3. Discussion

The aim of the present study was to investigate the effect of the commercially available probiotic formulation Serobioma against the LPS-induced intestinal inflammatory damage. Moreover, we aimed to explore the role of MCs in gut inflammation and in the maintenance of the intestinal epithelium integrity.

Building appropriate in vitro models is essential for studying the physiological mechanisms that occur within tissues and organs. In fact, the in vitro approach provides an extremely regulated system and reproducible experimental conditions to study intestinal functions. 

Here, Transwell co-culture systems were employed to physiologically reproduce the compartmentalization between the gut lumen and the host. In particular, three different in vitro experimental models were set up: (i) HT-29 cells cultured on a semi-porous membrane of the Transwell insert, interfaced with HMC-1.2 in the basolateral side; (ii) a co-culture model consisting of Caco-2 and HT-29 intestinal human cells, grown on a semi-porous membrane of the Transwell insert and interfaced with HMC-1.2 in the basolateral chamber; and (iii) an HT-29 and Caco-2 co-culture, in the absence of HMC-1.2 in the basolateral compartments, to test the potential ability of epithelial cells to secrete proinflammatory cytokines when stimulated with LPS.

HT-29 cells grow as multilayers and fail to generate stable tight junctions, as confirmed by the low TEER value in both treated and untreated samples (Figure 1B). Moreover, this model is far too simple to simulate the intestinal absorptive epithelium. The poor barrier function probably allowed the direct stimulation of HMC-1.2 with LPS/probiotics. On the contrary, in the Caco-2/HT-29 co-culture model, by using DAPI-staining we observed that HT-29 form clusters within the Caco-2 cell monolayer (Figure 5). However, unlike the Caco-2 cells, HT-29 are mucus-producing cells, and the co-culture is the best model to reproduce the physiological intestinal barrier [25,26].

The LPS induced the expected TEER reduction only in the absence of HMC-1.2 (Figure 1 and Figure 2), indeed, it has been reported to impair tight junction strength [28,29,30,31] via TLR4-dependent activation of the membrane-associated adaptor protein focal adhesion kinase in Caco-2 monolayers [32]. Interestingly, we showed that both the exposure to the probiotic formulation for 24 h, and the presence of HMC-1.2, concurred to preserve the integrity of the epithelial layer, as demonstrated by the TEER values in the Caco-2/HT-29 co-cultures. Moreover, HMC-1.2 did not alter the basal TEER value of the Caco-2/HT-29 layer (Figure 2).

These findings confirm that MCs are fundamental barrier elements for a physiological intercellular communication, and their presence can significantly influence the barrier function. Although MCs are generally discussed in the context of pathological conditions, it is important to reiterate that they also have a crucial role in homeostasis [33]. It has been reported that MCs regulate the intestinal epithelial permeability during the effector phase of intestinal inflammatory responses, modulating the expression and the distribution of junction proteins, and the intestinal architecture [34].

In the HMC-1.2 interfaced with HT-29, LPS stimulation induced a significant increase in IL-6 levels, while the probiotic formulation was able to prevent the LPS-induced release of IL-6 from HMC-1.2, at all concentrations tested. The results obtained suggest that even in the presence of intestinal barrier damage, such as in a leaky gut, the probiotic formulation is able to directly modulate the secretion of IL-6.

In the Caco-2/HT-29/HMC-1.2 co-culture model, the concentration of IL-6 released in the basal medium was higher than in the HT-29/HMC-1.2 model. To exclude that IL-6 was produced by Caco-2 cells, the same experimental protocol was applied in a Caco-2/HT-29 co-culture in the absence of HMC-1.2. The results showed that no release of IL-6 occurred. This experiment suggested that the production of IL-6 could be attributed only to MCs, and that Caco-2 cells, much more than HT-29, communicate with HMC-1.2 cells, stimulating the basal release of IL-6. This was confirmed by RT-qPCR. Indeed, we found a higher amount of *IL-6* mRNA in the HMC-1.2 interfaced with the Caco-2/HT-29 co-culture, than in the MCs seeded below the HT-29 (Figure 4).

The crosstalk between the Caco-2/HT-29 co-culture and HMC-1.2 could also influence the TNF-α release. As shown in Figure 2, the release of TNF-α by MCs after 4 h-LPS exposure without recovery was higher when cells were interfaced with the Caco-2/HT-29 co-culture. In the same model, the decrease in TNF-α from MCs after LPS exposure could be attributed to the temporary interruption of the crosstalk between the Caco-2/HT-29 cells and the HMC-1.2, and the establishment of an inflammatory status in the intestinal epithelial cells. Interestingly, the probiotic mixture at a 1:10 cell/CFU ratio was capable of counteracting the LPS effect on the TNF-α release, supporting a potential therapeutic role for probiotics. This is in agreement with evidence advising the use of specific probiotics in other inflammatory disorders [35].

These findings match with other in vitro evidence showing the existence of a mutual communication between MCs and colon cancer Caco-2 and HT-29 cells [36]. Similarly, crosstalk between epithelial cells and macrophages has been shown to be able to create an immunoregulatory microenvironment necessary for maintaining tissue homeostasis [37].

The multi-strain probiotic formulation tested in this study contained *L. rhamnosus* LR 32, *B. lactis* BL04, and *B. longum* BB 536. Lactobacillus strains were effective in modulating commensal microorganisms in a beneficial sense, and in inhibiting the adhesion of pathogens to the intestinal mucosa. The probiotics were shown to have positive immune balance remedial effects, and to help establish conditions of immune tolerance. It is now widely recognized that bacteria belonging to the probiotic genera of Bifidobacteria and Lactobacilli can participate in immune regulation [38,39,40]. Specific probiotic strains have been shown to stabilize MCs, in particular *L. rhamnosus* GG [41]. Moreover, oral administration of *L. rhamnosus* JB-1 induces inhibition of peritoneal MCs degranulation [42], while *L. rhamnosus* LR 32 exhibit immunomodulatory properties in in vitro models [43,44,45,46]. *Bifidobacterium longum subsp. longum* BB536 is a probiotic bacterial strain whose efficacy is now consolidated and widely known. It is used in gastrointestinal, immunological, and infectious diseases. In particular, it modulates luminal metabolism, stabilizing the intestinal microbiota [47], and it is able to modulate the immune response in elderly patients if administered prior to the influenza vaccine [48]. Furthermore, this strain can stabilize tight junction proteins through exopolysaccharide and the production of butyrate, as active metabolites [28,49]. The influence of probiotics toward MCs was also demonstrated for the *B longum* KACC 91563, that induces MCs’ apoptosis [50], and for the probiotic formulation VSL#3, capable of reducing MC degranulation [51].

Synergistic or additive actions may be seen in a multi-strain probiotic composition; this may be due to trophic cooperation between the strains, in which the by-products of one microorganism provide food for another. Furthermore, a bacterial strain can affect the environment in which it lives, impacting the proliferation of other bacterial species, preventing pathogen colonization [52]. Previous studies from in vitro and ex vivo experiments suggested that Serobioma accelerates the anti-inflammatory process, inducing a significant downregulation of the proinflammatory cytokines IL-1β and Il-6. In particular, it was shown that the probiotic formulation was able to modulate indirectly the immune proinflammatory response of macrophages through the epithelial cell monolayer [24]. Furthermore, the ability of the tested probiotic formulation to prevent LPS-induced damage toward the intestinal epithelial layer was demonstrated when used in pre-treatment, prior to the LPS exposure [28].

Taken together, our findings demonstrate that the probiotic mixture is able to regulate the activation of MCs through the intestinal epithelial barrier.

## 4. Materials and Methods

### 4.1. Cell Lines

Human colorectal adenocarcinoma Caco-2 and HT-29 cells were cultured as previously reported [28,53]. HMC-1.2 cells (HMC-1.2, Merck-Millipore, Darmstadt, Germany) were plated at 2.5 × 10^5^ cells/mL density in T25 flasks with Isove Medium, 1.2 mM α-thioglycerol (Sigma Cat No. M6145-100ML), 10% FBS (EMD Millipore Cat. No. ES-009-B) and 1× penicillin/streptomycin, and incubated at 37 °C in a humidified atmosphere with 5% CO_2_. The medium was replenished every 2–3 days and the cells were sub-cultured when the cell density was at 1–1.5 × 10^6^ cells/mL.

### 4.2. Transwell Experimental Models

#### 4.2.1. HT-29 Cell Layer Interfaced with HMC-1.2 Cells

HT-29 cells were seeded at the concentration of 6 × 10^4^ cells/well on a 24-well polycarbonate insert (6 mm diameter, 0.4 μm pore size). The cultures were maintained for 7 days before the experiment, in order to achieve full confluence. The medium was changed every two or three days [24]. Prior to the experiments, HMC-1.2 cells were suspended in fresh medium without antibiotics at 2.5 × 10^5^ cells/mL concentration, and 1 mL of cell suspension was placed in the basolateral chamber of the 24-well plate, while the Transwell inserts containing the confluent HT-29 cells were picked, and transferred onto the HMC-1.2.

#### 4.2.2. Caco2/HT-29 Cell Layer Interfaced with HMC-1.2 Cells

Caco-2 and HT-29 cells were seeded at the concentration of 8 × 10^4^ cells/well, on a 24-well polycarbonate insert at a 9:1 ratio [25]. Culture medium was replaced between 6 and 16 h after seeding to avoid multiple layers of Caco-2 cells forming [28,54]. The cultures were maintained for between 18 and 21 days before the experiment, to achieve complete differentiation of the Caco-2 cells. The medium was changed every two or three days. Prior to the experiments, HMC-1.2 cells were suspended in fresh medium without antibiotics at 2.5 × 10^5^ cells/mL concentration, and 1 mL of cell suspension was placed in the basolateral chamber of the 24-well plate, while the Transwell inserts containing the Caco-2/HT-29 cells were picked, and transferred onto the basolateral chamber.

#### 4.2.3. Caco-2/HT-29 Cell Layer

The Caco-2/HT-29 co-culture layer was established as described above.

**Figure 6:**
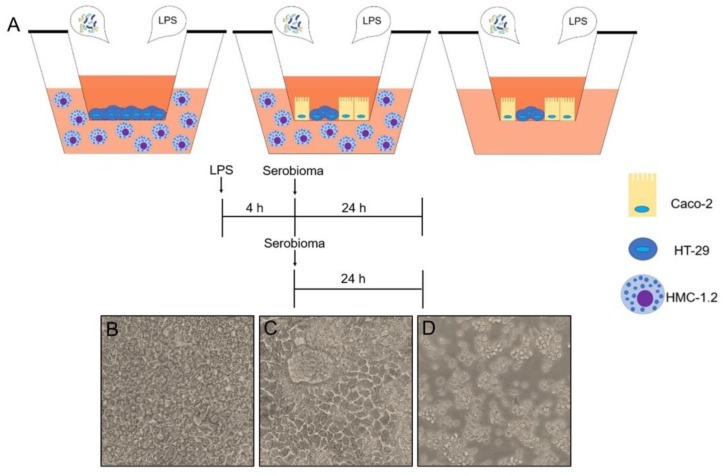
Schematic representation of the Transwell co-culture models (**A**). Microphotograph of 7-day confluent HT-29 (**B**), 18-day Caco-2/HT-29 co-culture (**C**), and HMC-1.2 (**D**), 20× magnification.

Prior to the experiment, the Transwell inserts containing Caco-2/HT-29 cells, were transferred onto the basolateral chamber containing 1 mL of HMC-1.2 fresh medium without cells. The Transwell experimental models are depicted in the Figure 6.

### 4.3. Treatment with the Probiotic Formulation

The commercial product Serobioma (Bromatech, S.r.l., Milan, Italy) contains *L. rhamnosus* LR 32, *B. lactis* BL 04, and *B. longum* BB 536.

Twenty-four hours prior to the experiment, cell medium—both for intestinal and mast cell lines—was replaced with fresh medium without antibiotics, and the latter was used throughout all the experimental procedure.

In this study, two different cells/CFU ratios were used, namely 1:1 (2.5 × 10^5^ CFU), and 1:10 (2.5 × 10^6^ CFU), with respect to HMC-1.2 cells. These concentrations were chosen based on previous experiments [17], and correlate with the number of viable probiotics that reach the human gut after the probiotic administration.

Before the experiment, HMC-1.2 were suspended in fresh medium and seeded in the basolateral side of the Transwell system as reported above. Thus, culture medium from the apical side was removed and replaced with fresh medium or medium containing LPS from *E. coli* 026:B6 (1 μg/mL). The plates were incubated in a humidified atmosphere, at 37 °C with 5% CO_2_. After 4 h LPS-incubation, apical medium was replaced with fresh medium or medium containing fresh bacterial suspension at the desired concentration. The cells were further incubated for 24 h with the probiotic formulation.

At the end of the treatment, the medium and HMC-1.2 cells were collected from the basolateral side. The cell suspension was centrifuged at 500× *g* for 10 min at 4 °C. The supernatants were filtered with a 0.22 μm pore syringe filter in order to remove cells and bacterial residues, and were stored at −80 °C until the cytokine determination. The cell pellets were washed with sterile phosphate buffered saline solution (PBS), and suspended in TRIzol^®^ reagent for RNA extraction.

At the same time, medium containing probiotics, in the apical side of the Transwell insert, was removed, and intestinal cell layers were washed with sterile PBS to remove probiotics adhered to the cells’ surfaces. Thus, 1 mL of HMC-1.2 fresh medium was placed in the basolateral side and 300 μL of Caco-2/HT-29 fresh medium was replenished in the apical side of the Transwell insert, in order to proceed to the TEER measurement.

### 4.4. TEER Measurement

The TEER measurement was performed using a Millicell-ERS Volt-Ohm meter (MilliporeSigma, Burlington, MA, USA) according to the manufacturer’s instructions. Briefly, electrodes were sterilized in 70% EtOH for 15 min and equilibrated for two hours in complete medium under a laminar-flow hood. The Volt-Ohm meter was calibrated using calibration electrodes. Three measurements were taken for each experimental point. The experiment was conducted in triplicate.

### 4.5. ELISA Assay

ELISA kits from Invitrogen (Thermo Fisher Scientific, Waltham, MA, USA) were used to quantify the IL-6 (KHC0061), IL-10 (KCH0101), IL-1β (BMS224-2), and TNFα (BMS223HS) cytokines released in the basolateral medium. All analyses were carried out according to the manufacturer’s kit instructions. For each cytokine measurement, dilution tests were previously conducted to identify the best concentration to obtain an optical density value within the required linearity range. Each sample was analyzed at least in duplicate, and a calibration curve was constructed for each test. The concentration of cytokines in each sample was calculated by interpolating the optical density to the calibration curve [54]. All experiments were carried out at least in triplicate.

### 4.6. RT-qPCR

RNA extraction from HMC-1.2 cells was conducted in accordance with the TRIzol reagent protocol. Then, 1 μg of RNA was reverse transcribed, using a High-Capacity cDNA Reverse Transcription Kit (Thermo Fisher Scientific, Waltham, MA, USA), in a 20 μL reaction mixture. The cDNA obtained was diluted at 1:5 and was amplified and quantified by RT-qPCR. The RT-qPCR reaction was carried out in a 20 μL mixture, containing 400 nM of forward and reverse primers (1.6 μL), 10 μL of ready-to-use PowerUP SYBR Green Master Mix (Thermo Fisher Scientific, Waltham, MA, USA), and RNase and DNase-free water (6.8 μL). As a reference gene, GAPDH was used. RT-qPCR reactions for each sample and gene were run in triplicate, and three independent experiments were carried out. The PCR conditions were 50 °C for 2 min, 95 °C for 10 min, and 40 cycles at 95 °C for 15 s and 60 °C for 1 min [55]. The sequences of primer pairs were as indicated in Table 1. The mRNA relative expression levels were calculated as 2^−ΔΔCt^.

### 4.7. DAPI Staining

In order to evaluate the cell growth on the Transwell system, after complete differentiation, the Caco-2/HT-29 co-culture was fixed and stained with DAPI. For the fixation, 300 μL of ice cold 4% formalin solution was added to each insert. The fixation was carried out for 5 min on ice, followed by two washes with PBS. The cells were permeabilised for 25 min using 300 μL of 0.5% Triton X-100 solution in PBS, and washed twice with PBS. Then, the insert was incubated for 5 min with 100 μL DAPI solution (10 μg/mL) in order to stain the cell nuclei. The insert membranes were detached from the insert housings using a scalpel and mounted onto microscopy slides. The nuclei were observed using an epi-fluorescent microscope (Olympus BX41, Tokyo, Japan), under a 100 W high-pressure mercury lamp (HSH-1030-L, Ushio, Japan), at 20× magnification.

### 4.8. Statistical Analysis

The statistical analyses were carried out using SPSS (SPSS Inc., Chicago, IL, USA). The data normal distribution was assessed by the Shapiro–Wilk test and a comparison between the treated and untreated samples was then performed by one-way ANOVA. The cells pre-incubated with LPS, and then with Serobioma, were also compared with the LPS-treated group. Moreover, The *IL-6* expression in HMC-1.2, when interfaced with the Caco-2/HT-29 co-culture or HT-29, was compared using the Student’s *t*-test. The level of significance was set at *p*-value < 0.05 for all experiments.

## 5. Conclusions

The results reported in the present study are part of a broader research work involving the Serobioma probiotic formulation. Treatment with this formulation is capable of reducing the inflammatory response in co-culture models of the intestinal barrier. Furthermore, it affects the crosstalk between intestinal epithelial cells and MCs.

Therefore, the specific probiotic supplementation could represent a promising therapeutic approach for the treatment of gut inflammatory diseases.

## Figures and Tables

**Table 1:** Primer pair sequences used for the RT-qPCR.

Accession Number	Gene Name	Symbol	Product Length	Primer Sequences (F: Forward; R: Reverse)
NM_002046.7	Glyceraldehyde-3- phosphate Dehydrogenase	*GAPDH*	120	F: TGACTTCAACAGCGACACCCA R: CACCCTGTTGCTGTAGCCAAA
NM_000600.5	Interleukin 6	*IL-6*	149	F: ACTCACCTCTTCAGAACGAATTG R: CCATCTTTGGAAGGTTGAGGTT

## Data Availability

All data are included in the manuscript.

## References

[1] Thursby E., Juge N. (2017). Introduction to the Human Gut Microbiota. Biochem. J..

[2] Ahluwalia B., Magnusson M.K., Öhman L. (2017). Mucosal Immune System of the Gastrointestinal Tract: Maintaining Balance between the Good and the Bad. Scand. J. Gastroenterol..

[3] Kong S., Zhang Y.H., Zhang W. (2018). Regulation of Intestinal Epithelial Cells Properties and Functions by Amino Acids. BioMed Res. Int..

[4] Garcia-Gutierrez E., Mayer M.J., Cotter P.D., Narbad A. (2019). Gut Microbiota as a Source of Novel Antimicrobials. Gut Microbes.

[5] Snoeck V., Goddeeris B., Cox E. (2005). The Role of Enterocytes in the Intestinal Barrier Function and Antigen Uptake. Microbes Infect..

[6] Traina G. (2019). Mast Cells in Gut and Brain and Their Potential Role as an Emerging Therapeutic Target for Neural Diseases. Front. Cell. Neurosc..

[7] Traina G. (2021). The Role of Mast Cells in the Gut and Brain. J. Integr. Neurosci..

[8] Kitamura Y., Ito A. (2005). Mast Cell-Committed Progenitors. Proc. Natl. Acad. Sci. USA.

[9] Dahlin J.S., Hallgren J. (2015). Mast Cell Progenitors: Origin, Development and Migration to Tissues. Mol. Immunol..

[10] Komi D.E.A., Wöhrl S., Bielory L. (2020). Mast Cell Biology at Molecular Level: A Comprehensive Review. Clin. Rev. Allergy Immunol..

[11] Frossi B., Mion F., Sibilano R., Danelli L., Pucillo C.E.M. (2018). Is It Time for a New Classification of Mast Cells? What do We Know about Mast Cell Heterogeneity?. Immunol. Rev..

[12] Beil W.J., Schulz M., Wefelmeyer U. (2000). Mast Cell Granule Composition and Tissue Location—A Close Correlation. Histol. Histopathol..

[13] Abraham S.N., St John A.L. (2010). Mast Cell-Orchestrated Immunity to Pathogens. Nat. Rev. Immunol..

[14] Supajatura V., Ushio H., Nakao A., Akira S., Okumura K., Ra C., Ogawa H. (2002). Differential Responses of Mast Cell Toll-Like Receptors 2 and 4 in Allergy and Innate Immunity. J. Clin. Investig..

[15] Thaiss C.A., Zmora N., Levy M., Elinav E. (2016). The Microbiome and Innate Immunity. Nature.

[16] De Zuani M., Dal Secco C., Frossi B. (2018). Mast Cells at the Crossroads of Microbiota and IBD. Eur. J. Immunol..

[17] Toor D., Wasson M.K., Kumar P., Karthikeyan G., Kaushik N.K., Goel C., Singh S., Kumar A., Prakash H. (2019). Dysbiosis Disrupts Gut Immune Homeostasis and Promotes Gastric Diseases. Int. J. Mol. Sci..

[18] Dominici L., Moretti M., Villarini M., Vannini S., Cenci G., Zampino C., Traina G. (2011). In vivo Antigenotoxic Properties of a Commercial Probiotic Supplement Containing Bifidobacteria. Int. J. Probiot. Prebiot..

[19] Dominici L., Villarini M., Trotta F., Federici E., Moretti G.C. (2014). and M. Protective Effects of Probiotic *Lactobacillus Rhamnosus* IMC501 in Mice Treated with PhIP. J. Microbiol. Biotechnol..

[20] Persichetti E., De Michele A., Codini M., Traina G. (2014). Antioxidative Capacity of *Lactobacillus Fermentum* LF31 Evaluated in vitro by Oxygen Radical Absorbance Capacity Assay. Nutrition.

[21] Traina G., Menchetti L., Rappa F., Casagrande-Proietti P., Barbato O., Leonardi L., Carini F., Piro F., Brecchia G. (2016). Probiotic Mixture Supplementation in the Preventive Management of Trinitrobenzenesulfonic Acid-Induced Inflammation in a Murine Model. J. Biol. Regul. Homeost. Agents.

[22] Bäckhed F., Fraser C.M., Ringel Y., Sanders M.E., Sartor R.B., Sherman P.M., Versalovic J., Young V., Finlay B.B. (2012). Defining a Healthy Human Gut Microbiome: Current Concepts, Future Directions, and Clinical Applications. Cell Host Microbe.

[23] Wouters M.M., Vicario M., Santos J. (2016). The Role of Mast Cells in Functional GI Disorders. Gut.

[24] Sichetti M., De Marco S., Pagiotti R., Traina G., Pietrella D. (2018). Anti-Inflammatory Effect of Multistrain Probiotic Formulation (*L. Rhamnosus*, *B. Lactis*, and *B. Longum*). Nutrition.

[25] Pan F., Han L., Zhang Y., Yu Y., Liu J. (2015). Optimization of Caco-2 and HT29 Co-Culture in vitro Cell Models for Permeability Studies. Int. J. Food Sci. Nutr..

[26] Srinivasan B., Kolli A.R., Esch M.B., Abaci H.E., Shuler M.L., Hickman J.J. (2015). TEER Measurement Techniques for in vitro Barrier Model Systems. J. Lab. Autom..

[27] Nilsson G., Blom T., Kusche-Gullberg M., Kjellén L., Butterfield J.H., Sundström C., Nilsson K., Hellman L. (1994). Phenotypic Characterization of the Human Mast-Cell Line HMC-1. Scand. J. Immunol..

[28] di Vito R., Conte C., Traina G. (2022). A Multi-Strain Probiotic Formulation Improves Intestinal Barrier Function by the Modulation of Tight and Adherent Junction Proteins. Cells.

[29] He S., Guo Y., Zhao J., Xu X., Wang N., Liu Q. (2020). Ferulic Acid Ameliorates Lipopolysaccharide-Induced Barrier Dysfunction via MicroRNA-200c-3p-Mediated Activation of PI3K/AKT Pathway in Caco-2 Cells. Front. Pharmacol..

[30] Wei C.-X., Wu J.-H., Huang Y.-H., Wang X.-Z., Li J.-Y. (2022). *Lactobacillus Plantarum* Improves LPS-Induced Caco2 Cell Line Intestinal Barrier Damage via Cyclic AMP-PKA Signaling. PLoS ONE.

[31] Guo S., Al-Sadi R., Said H.M., Ma T.Y. (2013). Lipopolysaccharide Causes an Increase in Intestinal Tight Junction Permeability in vitro and in vivo by Inducing Enterocyte Membrane Expression and Localization of TLR-4 and CD14. Am. J. Pathol.

[32] Guo S., Nighot M., Al-Sadi R., Alhmoud T., Nighot P., Ma T.Y. (2015). Lipopolysaccharide Regulation of Intestinal Tight Junction Permeability is Mediated by TLR4 Signal Transduction Pathway Activation of FAK and MyD88. J. Immunol..

[33] Conte C., Sichetti M., Traina G. (2020). Gut–Brain Axis: Focus on Neurodegeneration and Mast Cells. Appl. Sci..

[34] Groschwitz K.R., Hogan S.P. (2009). Intestinal Barrier Function: Molecular Regulation and Disease Pathogenesis. J. Allergy Clin. Immunol..

[35] Resta-Lenert S., Barrett K.E. (2006). Probiotics and Commensals Reverse TNF-α– and IFN-γ–Induced Dysfunction in Human Intestinal Epithelial Cells. Gastroenterology.

[36] Yu Y., Blokhuis B., Derks Y., Kumari S., Garssen J., Redegeld F. (2018). Human Mast Cells Promote Colon Cancer Growth via Bidirectional Crosstalk: Studies in 2D and 3D Coculture Models. Oncoimmunology.

[37] Hyun J., Romero L., Riveron R., Flores C., Kanagavelu S., Chung K.D., Alonso A., Sotolongo J., Ruiz J., Manukyan A. (2014). Human Intestinal Epithelial Cells Express Interleukin-10 through Toll-Like Receptor 4-Mediated Epithelial-Macrophage Crosstalk. J. Innate Immun..

[38] Vitetta L., Vitetta G., Hall S. (2018). Immunological Tolerance and Function: Associations between Intestinal Bacteria, Probiotics, Prebiotics, and Phages. Front. Immunol..

[39] Yao S., Zhao Z., Wang W., Liu X. (2021). Bifidobacterium Longum: Protection against Inflammatory Bowel Disease. J. Immunol. Res..

[40] Tamaki H., Nakase H., Inoue S., Kawanami C., Itani T., Ohana M., Kusaka T., Uose S., Hisatsune H., Tojo M. (2016). Efficacy of Probiotic Treatment with *Bifidobacterium Longum* 536 for Induction of Remission in Active Ulcerative Colitis: A Randomized, Double-Blinded, Placebo-Controlled Multicenter Trial. Dig. Endosc..

[41] Oksaharju A., Kankainen M., Kekkonen R.A., Lindstedt K.A., Kovanen P.T., Korpela R., Miettinen M. (2011). Probiotic *Lactobacillus Rhamnosus* Downregulates FCER1 and HRH4 Expression in Human Mast Cells. World J. Gastroenterol..

[42] Forsythe P., Wang B., Khambati I., Kunze W.A. (2012). Systemic Effects of Ingested *Lactobacillus Rhamnosus*: Inhibition of Mast Cell Membrane Potassium (IKCa) Current and Degranulation. PLoS ONE.

[43] Foligne B., Zoumpopoulou G., Dewulf J., Younes A.B., Chareyre F., Sirard J.-C., Pot B., Grangette C. (2007). A Key Role of Dendritic Cells in Probiotic Functionality. PLoS ONE.

[44] Kayama H., Takeda K. (2020). Manipulation of Epithelial Integrity and Mucosal Immunity by Host and Microbiota-Derived Metabolites. Eur. J. Immunol..

[45] Vale G.C., Mota B.I.S., Ando-Suguimoto E.S., Mayer M.P.A. (2021). Effect of Probiotics *Lactobacillus Acidophilus* and *Lacticaseibacillus Rhamnosus* on Antibacterial Response Gene Transcription of Human Peripheral Monocytes. Probiot. Antimicrob. Proteins.

[46] Vale G.C., Mayer M.P.A. (2021). Effect of Probiotic *Lactobacillus rhamnosus* by-Products on Gingival Epithelial Cells Challenged with *Porphyromonas Gingivalis*. Arch. Oral Biol..

[47] Wong C.B., Odamaki T., Xiao J. (2019). Beneficial Effects of *Bifidobacterium Longum* Subsp. Longum BB536 on Human Health: Modulation of Gut Microbiome as the Principal Action. J. Funct. Foods.

[48] Akatsu H., Iwabuchi N., Xiao J.-Z., Matsuyama Z., Kurihara R., Okuda K., Yamamoto T., Maruyama M. (2013). Clinical Effects of Probiotic *Bifidobacterium Longum* BB536 on Immune Function and Intestinal Microbiota in Elderly Patients Receiving Enteral Tube Feeding. JPEN J. Parenter. Enter. Nutr..

[49] Zhong Y., Wang S., Di H., Deng Z., Liu J., Wang H. (2022). Gut Health Benefit and Application of Postbiotics in Animal Production. J. Anim. Sci. Biotechnol..

[50] Kim J.-H., Jeun E.-J., Hong C.-P., Kim S.-H., Jang M.S., Lee E.-J., Moon S.J., Yun C.H., Im S.-H., Jeong S.-G. (2016). Extracellular Vesicle–Derived Protein from *Bifidobacterium Longum* Alleviates Food Allergy through Mast Cell Suppression. J. Allergy Clin. Immunol..

[51] Li Y.-J., Li J., Dai C. (2020). The Role of Intestinal Microbiota and Mast Cell in a Rat Model of Visceral Hypersensitivity. J. Neurogastroenterol. Motil..

[52] Moens F., Duysburgh C., van den Abbeele P., Morera M., Marzorati M. (2019). *Lactobacillus Rhamnosus* GG and *Saccharomyces Cerevisiae Boulardii* Exert Synergistic Antipathogenic Activity in Vitro against Enterotoxigenic *Escherichia coli*. Benef. Microbes.

[53] Villarini M., Acito M., di Vito R., Vannini S., Dominici L., Fatigoni C., Pagiotti R., Moretti M. (2021). Pro-Apoptotic Activity of Artichoke Leaf Extracts in Human HT-29 and RKO Colon Cancer Cells. Int. J. Environ. Res. Public Health.

[54] Hubatsch I., Ragnarsson E.G.E., Artursson P. (2007). Determination of Drug Permeability and Prediction of Drug Absorption in Caco-2 Monolayers. Nat. Protoc..

[55] Taticchi A., Urbani S., Albi E., Servili M., Codini M., Traina G., Balloni S., Patria F.F., Perioli L., Beccari T. (2019). In vitro Anti-Inflammatory Effects of Phenolic Compounds from Moraiolo Virgin Olive Oil (MVOO) in Brain Cells via Regulating the TLR4/NLRP3 Axis. Molecules.

